# Spontaneous Hemopneumothorax: An Uncommon Cause of Acute Hemodynamic Collapse

**DOI:** 10.7759/cureus.97099

**Published:** 2025-11-17

**Authors:** Anita Paiva, Ana Margarida Fernandes, Raquel Rodrigues, Florbela Oliveira, Pedro Fernandes

**Affiliations:** 1 Cardiothoracic Surgery, Unidade Local de Saúde de São João, Porto, PRT; 2 General Surgery, Unidade Local de Saúde da Póvoa de Varzim e Vila do Conde, Póvoa de Varzim, PRT

**Keywords:** acute-onset chest pain, hemodynamic instability (hdi), hypovolemic shock, spontaneous hemopneumothorax, urgent non cardiac surgery, video-assisted thoracic surgery

## Abstract

Spontaneous hemopneumothorax (SHP) is a rare but potentially life-threatening condition that may present with hemodynamic instability or even hypovolemic shock without an apparent cause. Given its nonspecific presentation, diagnosis is often overlooked or delayed. There are no standardized guidelines for SHP management, although the literature tends to favor early surgical intervention. Patients with hemodynamic instability, massive bleeding, persistent air leak, retained hemothorax, or trapped lung should undergo surgery. Minimally invasive surgery may be feasible in unstable patients who respond to fluid resuscitation. We report a case of a 21-year-old male patient who presented with sudden right-sided chest pain and unexplained hemodynamic instability. On admission, he was stable, and chest radiography showed a right pneumothorax with a moderate pleural effusion. A chest tube drained over two liters of blood. While awaiting a CT scan, the patient developed hypotension and tachycardia, prompting transfer to a tertiary hospital. Chest CT revealed active bleeding near the right subclavian artery. The patient underwent urgent video-assisted thoracic surgery (VATS), which demonstrated a moderate hemothorax and active bleeding from an apical adhesion likely disrupted by a spontaneous pneumothorax. Hemostasis was achieved, and the postoperative course was uneventful. SHP should be considered in young patients presenting with spontaneous pneumothorax and unexplained hemodynamic instability. Early diagnosis and prompt management are essential to prevent life-threatening hemorrhage and long-term complications. VATS represents a safe and effective surgical approach, even in unstable patients who respond to fluid replacement therapy.

## Introduction

Spontaneous hemopneumothorax (SHP) is a rare but potentially life-threatening condition characterized by the simultaneous presence of air and blood in the pleural cavity in the absence of trauma or other causes. It is estimated to complicate 1-12% of spontaneous pneumothoraces and represents a cause of unexplained hemodynamic instability and even hypovolemic shock in patients, particularly in young men presenting with sudden onset chest pain [1-5].

Because of its nonspecific initial presentation, SHP can be easily overlooked. Prompt diagnosis and management are essential to prevent complications. However, no standardized guidelines exist regarding the optimal treatment approach. Conservative treatment may be appropriate for selected patients, whereas early surgical intervention can help prevent fibrothorax and trapped lung. In recent years, video-assisted thoracic surgery (VATS) has gained importance in the management of SHP due to its therapeutic advantages [3,6-8].

We report a case of SHP in a young male patient who presented with acute chest pain and unexplained hemodynamic instability. This case highlights the diagnostic challenges and underscores the importance of early recognition and appropriate management of this uncommon entity.

## Case presentation

A 21-year-old male smoker with no prior medical history presented to the emergency department with the sudden onset of right-sided chest pain that worsened with inspiration. No history of trauma was reported. On admission, the patient was normocardic and normotensive, with an oxygen saturation of 98% on room air. Pulmonary auscultation revealed decreased breath sounds in the lower right hemithorax.

**Figure 1:**
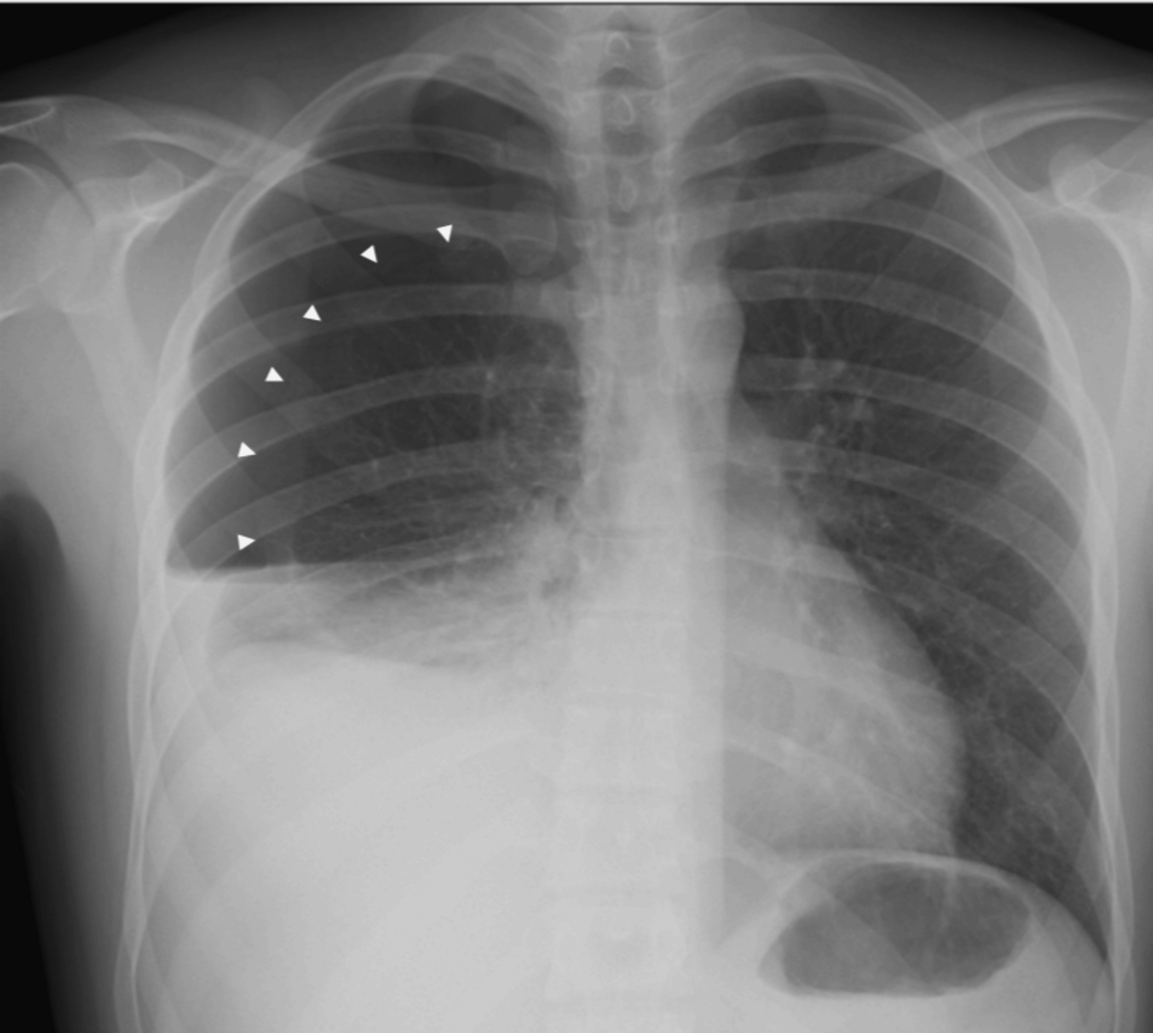
Chest radiograph at admission Posteroanterior chest radiograph demonstrating a right-sided pneumothorax with associated moderate pleural effusion. The lung is partially collapsed with a visible air-fluid level (white arrowheads outline the margin of the collapsed right lung).

Chest radiography showed a right-sided pneumothorax with a moderate pleural effusion (Figure 1). A right-sided chest tube was inserted, draining over one liter of blood. While awaiting a CT scan, the patient experienced diaphoresis and presyncope, associated with tachycardia and hypotension.

**Figure 2:**
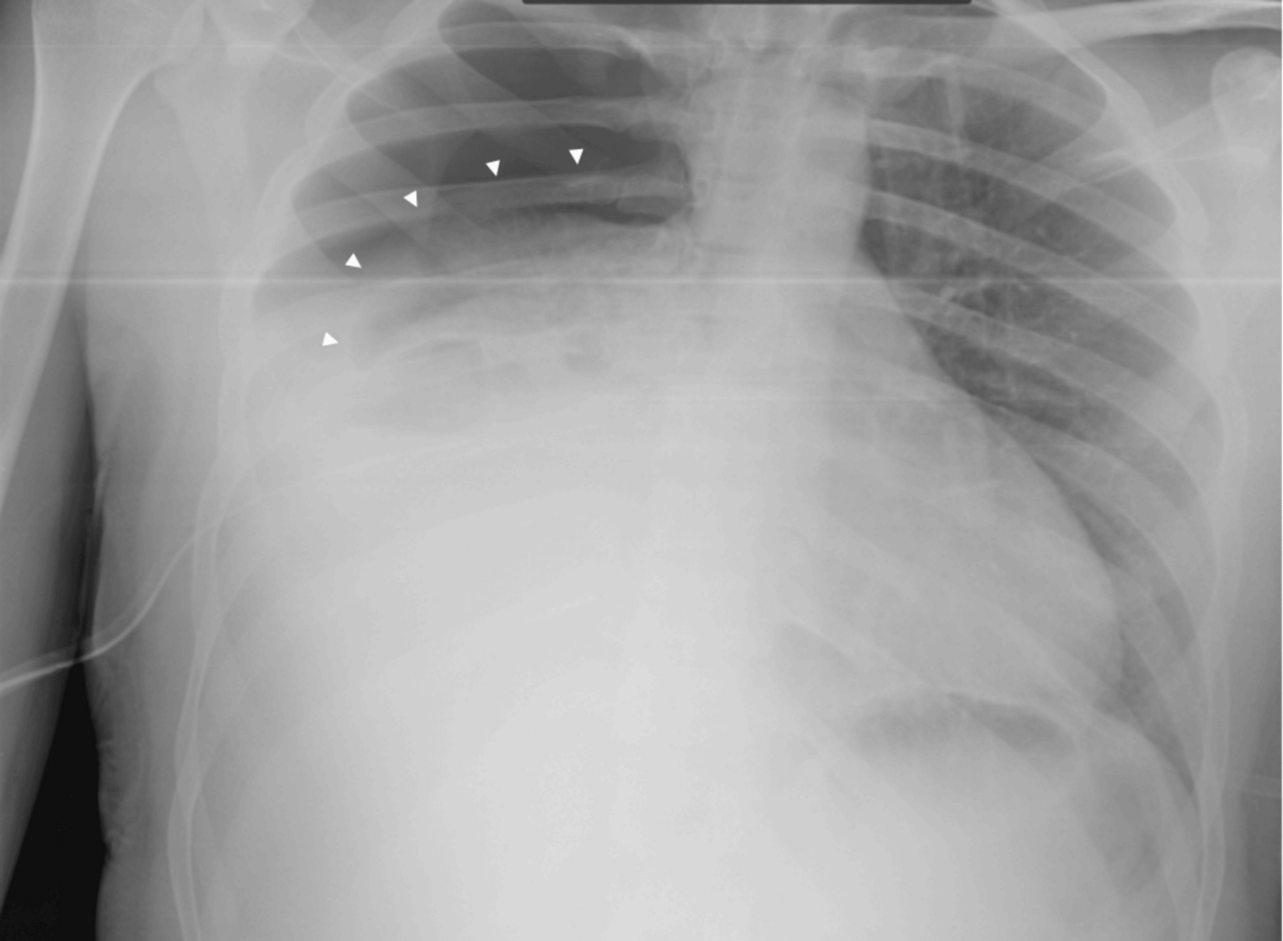
Post-drainage chest radiograph Radiograph after placement of a right-sided chest tube showing worsening of the pneumothorax and pleural effusion (white arrowheads outline the margin of the collapsed right lung).

Fluid resuscitation with isotonic crystalloids (normal saline) was initiated, and a repeat chest radiograph showed worsening of both the pneumothorax and the pleural effusion (Figure 2). At that point, hemoglobin had dropped 3 g/dL (Table 1). The patient showed clinical deterioration, with tachypnea and persistent tachycardia, and hypotension. Within the following hour, the chest tube drained an additional liter of blood, prompting the insertion of a second right-sided chest tube. As no CT scan was available at the facility, a thoracic surgeon was consulted, and the patient was transferred to a tertiary hospital.

**Table 1:** Laboratory findings at admission and on repeat testing (approximately two hours apart).

Parameter	At admission	On repeat	Normal range
Hemoglobin	14	10.8	13-18 g/dL
Hematocrit	39.2	31.2	43-55 %
White blood cell	15	19	4-11 x 10^9^/L
Neutrophils	79.5	83.1	53.8-69.8 %
Platelets	358	288	150-400 x 10^9^/L
Creatinine	0.82	0.92	0.67-1.17 mg/dL
C-reactive protein	3.5	14.7	< 3 mg/L

**Figure 3:**
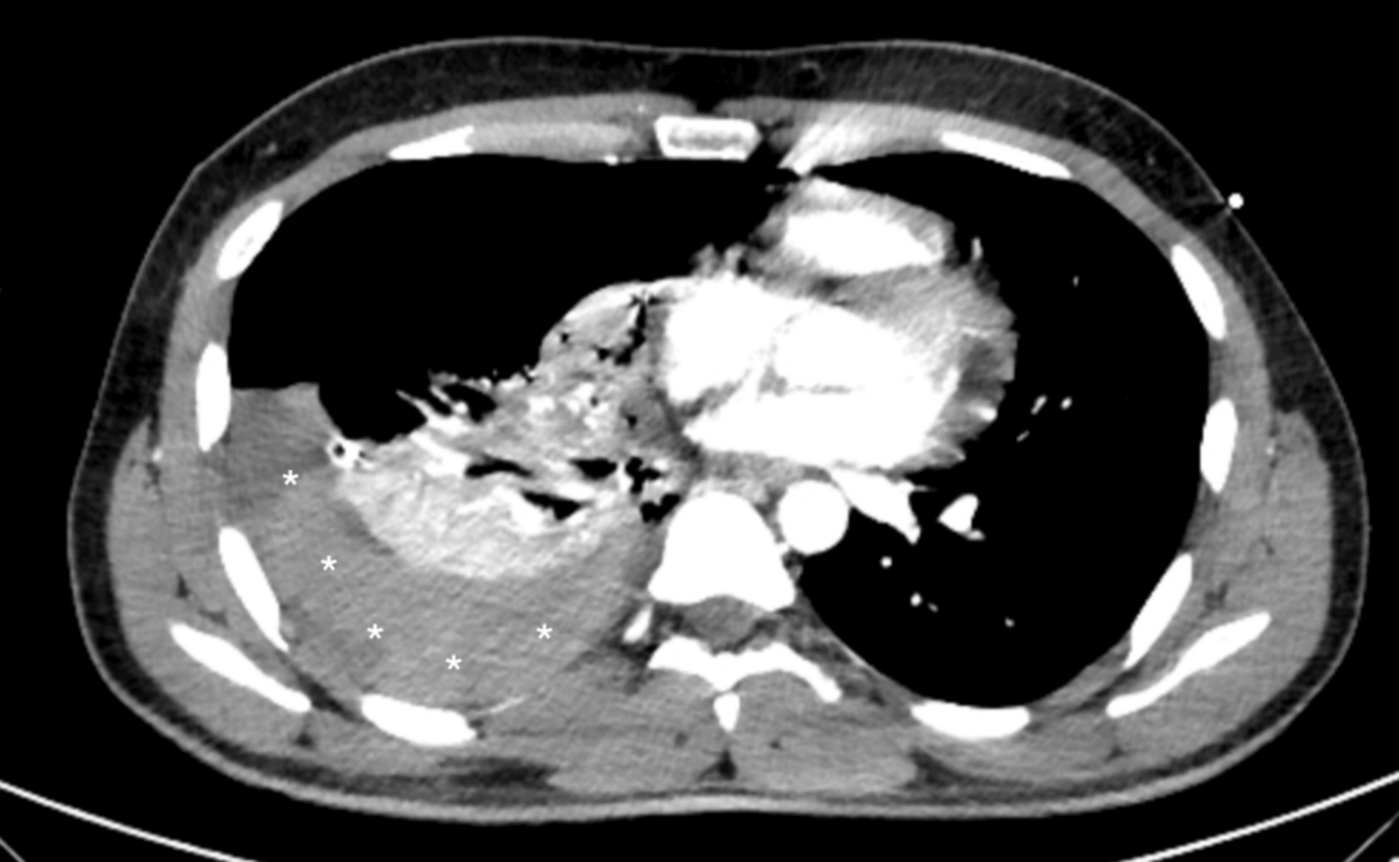
Axial contrast-enhanced chest CT demonstrating a moderate right-sided hemothorax Note: asterisks denote the area of pleural effusion

Upon admission, ongoing fluid resuscitation with normal saline and transfusion of one unit of packed red blood cells were administered to achieve hemodynamic stabilization. Chest CT demonstrated a right-sided moderate hemopneumothorax (Figure 3), with contrast enhancement distal to the medial third of the right subclavian artery during the arterial phase and a contrast blush in the right lung apex and pleural region suggestive of active bleeding.

**Figure 4:**
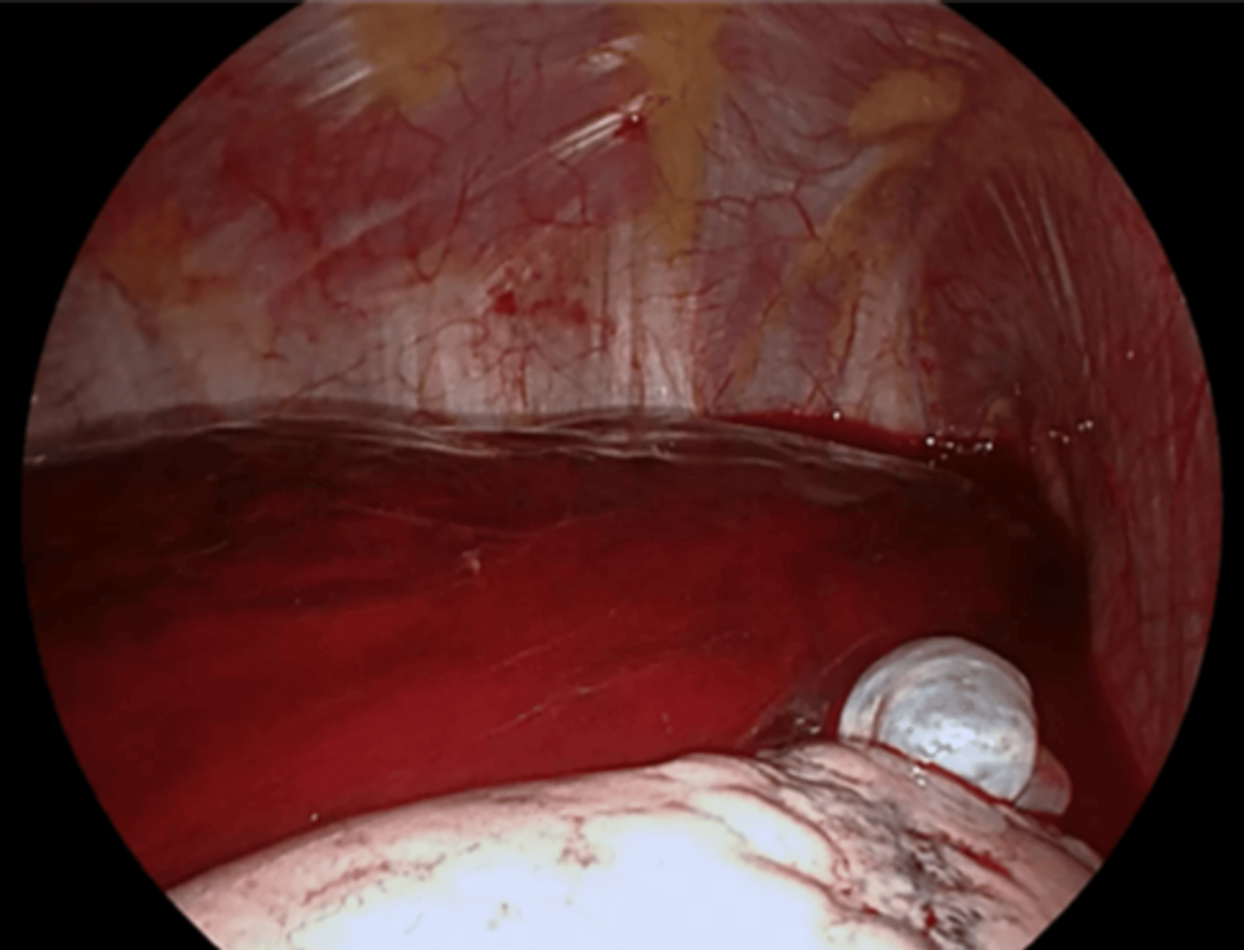
Intraoperative image showing a moderate hemothorax and apical pulmonary blebs

**Figure 5:**
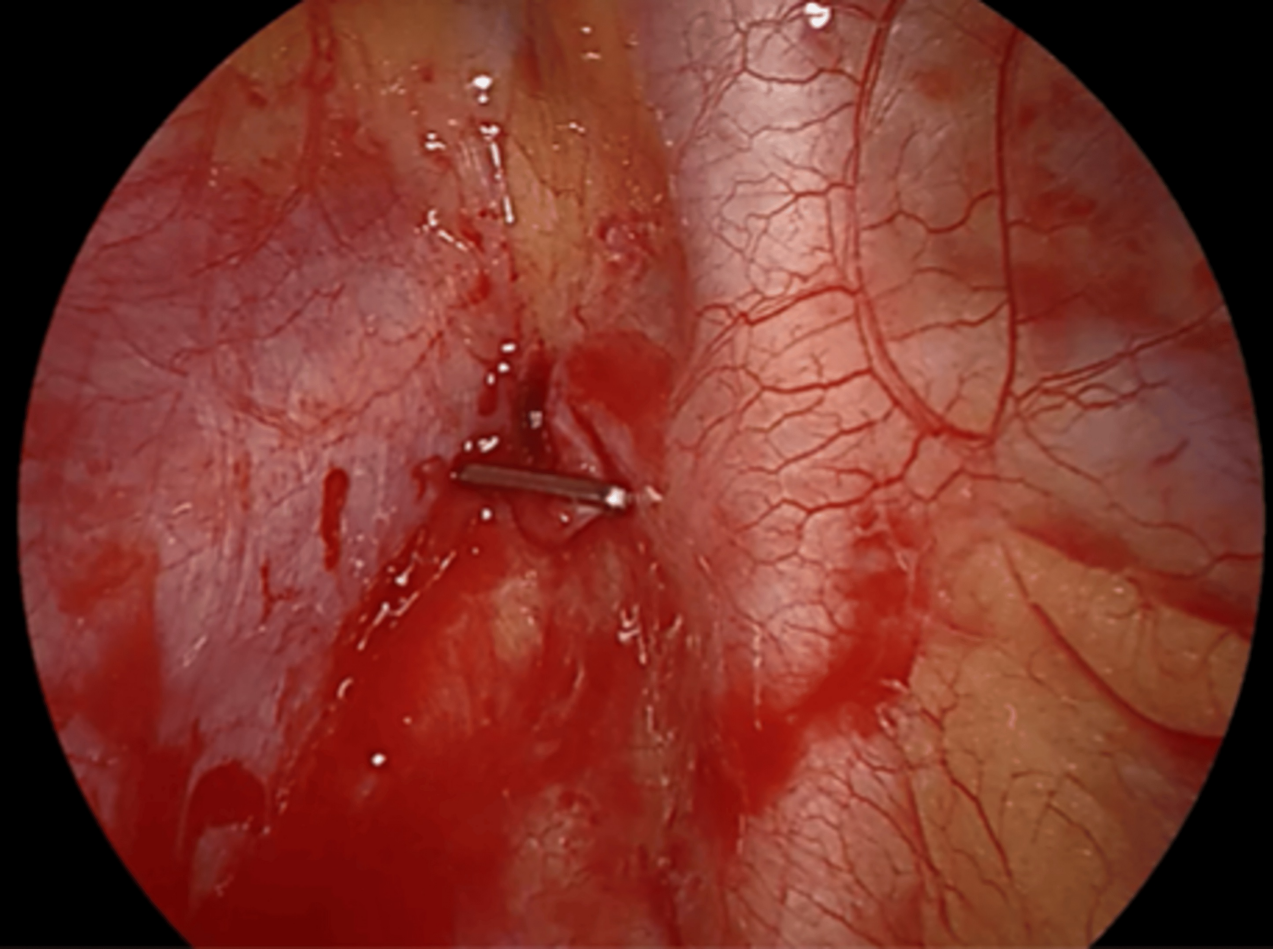
Intraoperative image showing a disrupted apical adhesion that was clipped during surgery

An urgent right uniportal VATS was performed. A moderate hemothorax was identified, along with pulmonary blebs in the right lung apex (Figure 4). Active bleeding was noted originating from the apical region, likely due to the disruption of an adhesion between the lung apex and the thoracic wall caused by a spontaneous pneumothorax. Hemostasis was achieved by clipping the disrupted adhesion (Figure 5). 

**Figure 6:**
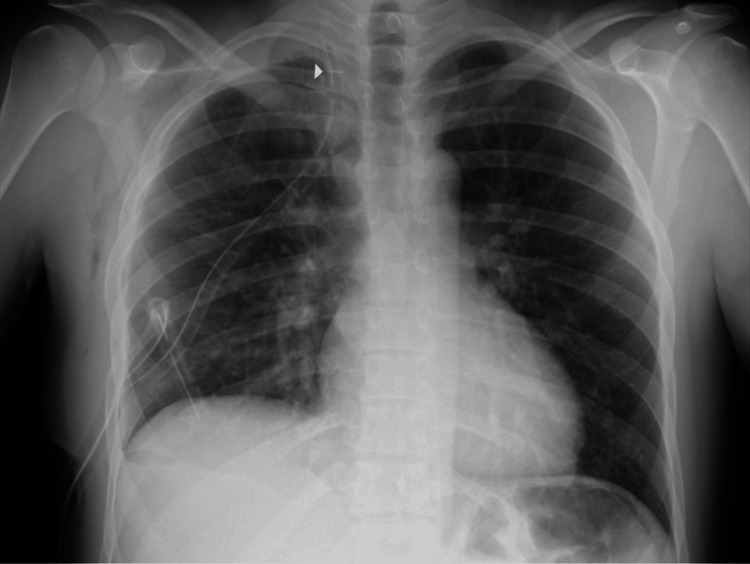
Postoperative chest radiograph Postoperative chest radiograph showing satisfactory re-expansion of the right lung with a small residual pleural effusion. The white arrowhead indicates the surgical clip applied to the disrupted apical adhesion during surgery.

Given the intraoperative blood loss, the patient received an additional unit of packed red blood cells. After drainage of the pleural cavity and a thorough inspection, no further signs of active bleeding were observed. The apical blebs were resected using a stapler, and pleurodesis was performed through pleural abrasion and talc insufflation. Two chest tubes were placed. Postoperative chest radiograph showed satisfactory re-expansion of the right lung with a small residual pleural effusion (Figure 6). The postoperative course was uneventful, and the patient was discharged on postoperative day four. After two years of follow-up, he remained asymptomatic with no recurrence of pneumothorax. 

## Discussion

SHP is a rare condition that complicates 1-12% of primary spontaneous pneumothorax (PSP). Similar to PSP, it exhibits a male predominance, and chest pain is the most frequent presenting symptom [1,2]. SHP is characterized by a simultaneous accumulation of air and blood in the pleural cavity in the absence of trauma or other causes. However, the definition of SHP remains unclear in the literature. While some authors consider SHP to involve the presence of at least 400 mL of blood in the pleural cavity in association with spontaneous pneumothorax [3], others define it by hemothorax of any volume accompanying a spontaneous pneumothorax [4].

Although some patients with SHP have no prior history of spontaneous pneumothorax, one theory that may explain this entity is that bleeding results from the rupture of aberrant vessels following the tearing of an apical vascularized adhesion. These vessels, most likely branches from the subclavian arteries, lie within adhesions between the visceral and the parietal pleura. Such adhesions are believed to develop after a previous minor bullous rupture or inflammatory process, since most bullae tend to develop in the apical regions of the lungs, near these major arteries. Other proposed mechanisms include rupture of a vascularized bulla and the underlying lung parenchyma or tearing of congenital aberrant vessels connecting the parietal pleura and bullae (thin-walled and lacking muscle fibers that allow adequate contractility in response to hemorrhage) [2,7,9,10].

Patients typically present with a sudden onset of chest pain and/or dyspnea secondary to spontaneous pneumothorax. If tearing of an apical vascularized adhesion occurs due to the presence of pneumothorax, the bleeding is not contained by the lung, allowing continued hemorrhage into the pleural cavity. This may rapidly progress to a life-threatening condition with hemodynamic instability and, in severe cases, hypovolemic shock [5].

A chest radiograph is the initial imaging modality of choice. The majority of patients with SHP will have an initial radiograph demonstrating a hydropneumothorax. However, physicians should be aware that approximately 10% of these patients may present only with a pneumothorax. This may be explained by delayed hemorrhage, an X-ray taken too early, or an X-ray obtained in the supine rather than the sitting or erect position. A chest CT scan with intravenous contrast can be performed if the diagnosis of SHP is uncertain or to rule out other causes of hemothorax [11].

In the diagnostic evaluation of SHP, it is essential to consider and exclude other potential causes of hemothorax, such as trauma, ruptured vascular malformations, pulmonary embolism with infarction, coagulopathies, or malignancy [3,5]. In this case, the absence of trauma or anticoagulant use, along with imaging findings consistent with rupture of an apical adhesion, supported the diagnosis of SHP.

The initial approach in hemodynamically stable patients involves chest tube placement. The presence of a hemothorax can be confirmed by a pleural fluid hematocrit greater than 50% of the patient’s blood hematocrit, which helps to distinguish it from a simple blood-stained effusion. The amount of blood drained is likely to underestimate the true blood loss due to the apical positioning of the chest tube, which is primarily intended to drain the pneumothorax, or because of tube obstruction by clot formation. Hemodynamic stability may also be misleading, as most patients with SHP are young and can tolerate significant hypovolemia before hemodynamic decompensation [11].

The management of SHP remains a matter of debate, ranging from conservative treatment to early or delayed surgical intervention. In hemodynamically stable patients in whom the bleeding ceases within 24 hours after chest tube placement, conservative treatment with tube thoracostomy and fluid resuscitation (including blood transfusion if necessary) may be sufficient to re-expand the lung, control the bleeding, and seal a potential air leak [12]. Conservative treatment can also be considered in patients without persistent air leak or evidence of trapped lung after hemopneumothorax drainage. Hemodynamically unstable patients or those with ongoing bleeding (> 100 mL per hour) should undergo emergent surgery. Delayed surgery is indicated for persistent air leak or retained hemothorax and can help resect bullae and prevent fibrothorax [3]. Some authors advocate early surgical intervention even in stable patients to prevent retained hemothorax, subsequent trapped lung, and the need for delayed decortication [6]. VATS offers several advantages over thoracotomy, as it provides excellent visualization of the entire pleural cavity and is associated with less postoperative pain, shorter hospital stay, and reduced blood loss. VATS also allows identification and control of the bleeding source, bullae resection, and, if necessary, pleurodesis [3,6-8]. In such cases, pleural abrasion should be considered over pleurectomy to minimize intraoperative bleeding. VATS may be an option in hemodynamically unstable patients who respond to fluid replacement therapy. However, in cases of hypovolemic shock or massive bleeding, thoracotomy remains the approach of choice [11,13].

In this case, early recognition of hemodynamic instability and prompt surgical management were crucial for a favorable outcome. The decision to transfuse, although the patient’s hemoglobin was not critically low, was guided by clinical judgement based on evidence of ongoing blood loss, persistent hemodynamic instability despite fluid resuscitation, and the anticipated hemodilution effect of intravenous fluids. In this clinical scenario, VATS proved to be the most appropriate surgical approach. Hemodynamic stabilization achieved after fluid resuscitation and blood transfusion allowed a minimally invasive procedure to be performed safely. VATS provided adequate exposure for identification and control of the bleeding source, as well as prevention of recurrent pneumothorax through bullae resection and pleurodesis, resulting in an excellent postoperative outcome. Thoracotomy may offer a wider field and better control in cases of massive bleeding or severe hemodynamic compromise; however, in this case, VATS achieved effective hemostasis with minimal morbidity, reinforcing its value even in selected unstable patients. This case also highlights the importance of individualized management in SHP.

## Conclusions

Although uncommon, SHP is a potentially life-threatening condition that should be considered as a cause of unexplained hemodynamic instability in patients presenting with spontaneous pneumothorax. Chest tube placement and fluid resuscitation remain the cornerstones of its management. Early surgical intervention may be indicated to prevent fibrothorax and delayed decortication. VATS is an option, even in hemodynamically unstable patients who respond to fluid replacement.
